# Survival and predictors of mortality among chronic kidney disease patients on hemodialysis in Amhara region, Ethiopia, 2021

**DOI:** 10.1186/s12882-022-02825-4

**Published:** 2022-05-23

**Authors:** Sewnet Getaye Workie, Taye Abuhay Zewale, Gizachew Tadesse Wassie, Makda Abate Belew, Eleni Dagnaw Abeje

**Affiliations:** 1 Department of Public Health, School of Public Health, College of Medicine and Health Science, Debre Berhan University, PO box 445, Debre Berhan, Ethiopia; 2 Department of Epidemiology and Biostatistics, School of Public Health, College of Medicine and Health Science, Bahir Dar University, PO box 79, Bahir Dar, Ethiopia; 3 Department of Nursing, School of Nursing and Midwifery, College of Medicine and Health Science, Debre Berhan University, PO box 445, Debre Berhan, Ethiopia

**Keywords:** Survival, CKD, Hemodialysis, Amhara region

## Abstract

**Background:**

Despite the high economic and mortality burden of chronic kidney disease, studies on survival and predictors of mortality among patients on hemodialysis in Ethiopia especially in the Amhara region are scarce considering their importance to identify some modifiable risk factors for early mortality to improve the patient’s prognosis. So, this study was done to fill the identified gaps. The study aimed to assess survival and predictors of mortality among end-stage renal disease patients on hemodialysis in Amhara regional state, Ethiopia, 2020/2021.

**Method:**

Institution-based retrospective record review was conducted in Felege Hiwot, Gonder, and Gambi hospitals from March 5 to April 5, 2021. A total of 436 medical records were selected using a simple random sampling technique. A life table was used to estimate probabilities of survival at different time intervals. Multivariable cox regression was used to identify risk factors for mortality.

**Result:**

Out of the 436 patients 153 (35.1%) had died. The median survival time was 345 days with a mortality rate of 1.89 per 1000 person-days (95%CI (1.62, 2.22)). Patients live in rural residences (AHR = 1.48, 95%CI (1.04, 2.12)), patients whose cause of CKD was hypertension (AHR = 1.49, 95%CI (1.01, 2.23)) and human immune virus (AHR = 2.22, 95%CI (1.41, 3.51)), and patients who use a central venous catheter (AHR = 3.15, 95%CI (2.08, 4.77)) had increased risk of death while staying 4 h on hemodialysis (AHR = 0.43, 95%CI (0.23, 0.80)) decreases the risk of death among chronic kidney disease patients on hemodialysis.

**Conclusions:**

The overall survival rate and median survival time of chronic kidney disease patients on hemodialysis were low in the Amhara region as compared with other developing Sub–Saharan African counties.

## Background

Although 1.9 million patients are undergoing renal replacement therapy (RRT) worldwide, yielding usage of 316 per million population, and annual initiation of 73 per million population, only about one-third (648,000) of the patients reside in developing regions, which contribute 85% of the world’s population [1]. There is also a high mortality rate of patients on hemodialysis, especially in the first 3 months after initiation of dialysis with annual mortality around 9% per year and with a 5-year survival of 40–50% [2].

In many developing countries, there is a shortage of renal replacement services which causes an estimated 2.3–7.1 million premature deaths [3]. African patients with ESRD have the lowest access to RRT with only 9–16% being treated; in central and eastern Africa, the treatment rate is estimated to be as low as 1–3% [3, 4].

Despite the establishment of renal registries and publication of data about RRT, there is a lack of countrywide publication in many African countries due to sustainability issues [5]. Consequently, less is known about initial survival or the risk factors which cause early mortality in dialysis patients. However, mortality during the first 90 days remains high, and modifications might be made during this critical period to impact not only early survival but also possibly long-term survival of patients on dialysis [6, 7].

Ethiopia like other developing countries struggles with a double burden of communicable and non-communicable diseases that contributes to the growing burden of ESRD, so there is a limitation of resources to address RRT to all patients who require it [8–10]. Since the first private dialysis center was opened in the early 2000s and the first public unit in 2013 dialysis is considered to be a service for the rich [10, 11]. The current Ethiopian health care system is structured into a three-tier system namely primary, secondary and tertiary level health care. Hemodialysis service is given at tertiary level health care and the main source to cover the expenses of the service is out of pocket money of the patients [10].

Inaccessibility of dialysis centers and financial constraints were the main reasons for delayed initiation of hemodialysis and frequent discontinuation in the country [10, 12]. Frequent breakdown of machines, electric power fluctuation, and lack of filtered water were also the main reasons for reduced sessions per week and reduced duration of hemodialysis per session from the standard [12]. In addition to the limited access to the service, quality is also compromised due to the limitation of duration and frequency of treatment, and the restriction of publicly funded dialysis for patients eligible for transplantation [10]. The cost of hemodialysis treatment among end-stage renal disease patients at the tertiary hospitals of Addis Ababa city and the Amhara region shows that the annual cost of the hemodialysis treatment was ETB 121,089.27 ($4466.59) per patient per year [13].

In Ethiopia, many prevalence studies on CKD have been done and show the increasing burden of the disease but only a few studies have been done to understand survival rates and predictors of mortality among CKD patients on hemodialysis [14–17]. The previous studies were conducted using relatively small sample size and single dialysis unit. In Amhara regional state there were no previous studies done on the same topic to date.

There are two governmental and one non-governmental hemodialysis center in the region namely; Felege Hiwot, the university of Gondar teaching referral hospitals, and Gambi teaching hospital. The Hospitals give service to nearly 800 patients. Felege Hiwot comprehensive specialized hospital and Gambi private teaching hospitals are found in Bahir Dar city. Gambi teaching hospital started hemodialysis service in 2014 while Felege Hiwot comprehensive specialized hospital started in 2015. University of Gondar teaching hospital is found in Gondar town, northwestern Ethiopia. The hospital provides inpatient and outpatient medical services in several departments. The hospital started hemodialysis service in 2017 [18].

The study was done to fill the identified gaps using multiple dialysis units to effectively assess the survival status and predictors of mortality among CKD patients on hemodialysis in Amhara region, Ethiopia.

## Methods and materials

An institution-based retrospective follow-up study was conducted among chronic kidney disease patients who undergo hemodialysis from March 1, 2016, to March 1, 2021, in the region. The medical record numbers of the patients from Felege Hiwot (385 medical records), Ghambi (200 medical records), and Gonder hospitals (215 medical records) were retrieved. Then by merging the registered medical numbers to create a sampling frame of 800 medical records, the 436 computer-generated random records were selected.

The dependent variable of the study is event i.e., death at time t (event = 1 and censored = 0) and the independent variables include socio-demographic factors, clinical factors, possible etiology of CKD, and presence of comorbidity.

The information available on the eligible patients’ medical records was collected using a data extraction tool prepared by adapting from different works of literature [14–17]. The selected charts of CKD patients on hemodialysis between March 01, 2016, to March 01, 2021, at Felege Hiwot, Gondar, and Gambi hospitals were retrieved and then reviewed. Baseline data of the patients were taken from the charts. Death certificates were extracted from the hospital registries by the patient’s medical record number. For those patients whose outcomes were not registered, it was confirmed using a phone call to their close family members.

In the current study censored means patients whose status was unknown (lost to follow-up), patients who did not develop the outcome of interest (death) until March 01/2021, those patients transferred or referred to other health institutions, and patients who died with an accident (e.g., car accidents). The event is the death of CKD patients on hemodialysis. The follow-up period was the time from the beginning of the study (March 01/ 2016) to an event, the end of the study (March 01/2021), or loss of contact or withdrawal from the study.

### Data processing and analysis

After the data collection, the collected data were entered into Epi data version 3.1 and then exported to STATA™ 14.1 for data cleaning and analysis. The life table was used to estimate probabilities of survival after initiation of hemodialysis at different time intervals and the cumulative probability of survival for each year. Kaplan Meier survival curves were used to compare the survival differences among categories of the variables.

Before running the Cox Proportional hazard regression model, the proportional hazard assumption was checked using global goodness of fit based on Schoenfeld residual, and variables having a *P*-value > 0.05 were considered as fulfilling the assumption. The *p*-value for the global test was 0.5661, and each predictor variable had a *p*-value greater than 0.05.

To identify potential predictors of mortality of ESRD patients on hemodialysis bi-variable Cox proportional regression model was fitted for each explanatory variable. Those having a *P* value less than 0.25 in the bi-variable analysis were included in the multivariable Cox proportional hazard regression model in the multivariable analysis. Variables having a *P* value less than 0.05 with 95% CI was considered significant predictors of time to death and an adjusted hazard ratio was used to show the strength of association between each predictor variable and the outcome variable. Finally, the result of the study was presented using text, tables, and graphs.

## Results

### Socio-demographic characteristics of the patients

Among 436 patients who participate in the study, the median age was 45 years (IQR 55 − 35 years). The number of male participants was 268 (61.5%). More than half of the participants live in urban residences 282 (64.7%). Most of the patients 365 (83.7%) had no family history of CKD (Table 1).

**Table 1:** Sociodemographic characteristics of CKD patients on hemodialysis in Amhara regional state, Ethiopia, 2016-21 (*n* = 436)

Variable categories		Frequency	Percent (%)
Gender	Female	168	38.5
	Male	268	61.5
Residence	Urban	282	64.7
	Rural	154	35.3
Family history	No	365	83.7
	Yes	71	16.3

### Etiology of end-stage renal diseases

Among 436 ESRD patients who were on hemodialysis, the primary causes of chronic kidney disease were 225(51.6%) hypertension, 130(29.8%) diabetes, 137(31.4%) glomerulonephritis, 31(7.1%) polycystic kidney disease, 47(10.8%) HIV and 35(8.0%) of the patients due to unknown causes as shown in Table 2.

**Table 2:** Primary causes of end stage renal disease among patients on hemodialysis Amhara regional state, Ethiopia,2016-21 (*n* = 436)

Variable categories		Frequency	Percent (%)
causes of end stage renal disease	Diabetes mellitus	130	29.8
	Hypertension	225	51.6
	Glomerulonephritis	137	31.4
	Polycystic kidney disease	31	7.1
	HIV	47	10.8
	Unknown/Idiopathic	35	8.0

Total will not add up to 436 or 100% as multiple responses were possible

### Presence of comorbidity

Among the 436 patients, 396(90.8%) had comorbidity. The leading comorbidity was hypertension 313(80.3%) followed by anemia 245(62.8%) and diabetes 112(28.7%) (Table 3).

**Table 3:** Types of comorbidities among ESRD patients on hemodialysis in Amhara regional state, Ethiopia, 2016-21 (*n* = 396)

Variable categories		Frequency	Percent (%)
Comorbid diseases among ESRD patients	Anemia	245	62.8
	Myocardial Infarction	27	6.9
	Congestive heart failure	104	26.7
	Hypertension	313	80.3
	Diabetes	112	28.7
	HIV/AIDS	60	15.4
	Cerebrovascular disease	23	5.9
	Cancer	13	3.3
	Peripheral vascular disease	21	5.4
	Infection	13	3.3

The result will not add up to 396 or 100% as multiple responses were possible

### Clinical characteristics of the patients

Among the 436 patients, 221(50.7%) used arteriovenous fistula, the other 171 (39.2%) and 44(10.1%) use a central venous catheter and arteriovenous graft respectively as their vascular access. About 331(75.9%) patients undergo hemodialysis twice, 73(16.7%) three times, and 32(7.3%) once per week. Regarding the duration of hemodialysis per session 378(86.7%) spend 4 h, 35(8.0%) for three and half hours and 23(5.3%) spend 3 h on hemodialysis per session (Table 4).

**Table 4:** Types of vascular access among ESRD patients on hemodialysis in Amhara regional state, Ethiopia, 2016-21 (*n* = 436)

Variable categories		Frequency (percent)
Vascular access	Fistula	221 (50.7%)
	Catheter	171 (39.2%)
	Graft	44 (10.1%)
Frequency per week	Once	32 (7.3%)
	Twice	331 (75.9%)
	Three times	73 (16.7%)
Duration per session	< 3 h.	23 (5.3%)
	3 and ½ hours	35 (8.0%)
	4 h	378 (86.7%)
Adequacy	< 1.2 kt/v	74 (17.0%)
	> 1.2 kt/v	362 (83.0%)
Blood transfusion	No	272 (62.4%)
	Yes	164 (37.6%)
Medication	No	75 (17.2%)
	Yes	361 (82.8%)

### Survival outcomes of ESRD patients on hemodialysis

Among 436 patients who were included in the study 153 (35.1%) (95%CI (30.7, 39.7)) had died during the 5 years follow-up time with a median survival time of 345 days (95%CI (278, 419)). The 436 patients contributed 80,774 person-days to the follow up making the incidence rate of death 1.89 (95%CI (1.61, 2.21)) per 1000 person-days.

The survival probability of the patients at three months was 85.46% (95%CI (81.61, 88.57)), and at 1 year it was 49.58% (95%CI (42.13, 56.58)) as shown in Table 5.

**Table 5:** Survival probability of ESRD patients on hemodialysis with 90 days interval in Amhara regional state, Ethiopia, 2016-21

Interval	Beg. Total	Deaths	Lost	Survival	Std. Error	[95% Conf. Int.]
[0–90]	436	58	74	0.8546	0.0176	0.82, 0.89
[90–180]	304	34	102	0.7398	0.0239	0.69, 0.78
[180–270]	168	25	54	0.6086	0.0308	0.54, 0.67
[270–360]	89	14	27	0.4958	0.0370	0.42, 0.56
[360–450]	48	7	16	0.4090	0.0427	0.32, 0.49
[450–540]	25	8	2	0.2727	0.0486	0.18, 0.37
[540–630]	15	0	4	0.2727	0.0486	0.18, 0.37
[630–720]	11	0	2	0.2727	0.0486	0.18, 0.37
[720–810]	9	2	2	0.2045	0.0554	0.11, 0.32
[810–900]	5	2	0	0.1227	0.0558	0.04, 0.25
[900–990]	3	1	0	0.0818	0.0500	0.02, 0.21
[990–1080]	2	1	0	0.0409	0.0382	0.003, 0.16
[1530–1620]	1	1	0	0.0000		

**Fig. 1:**
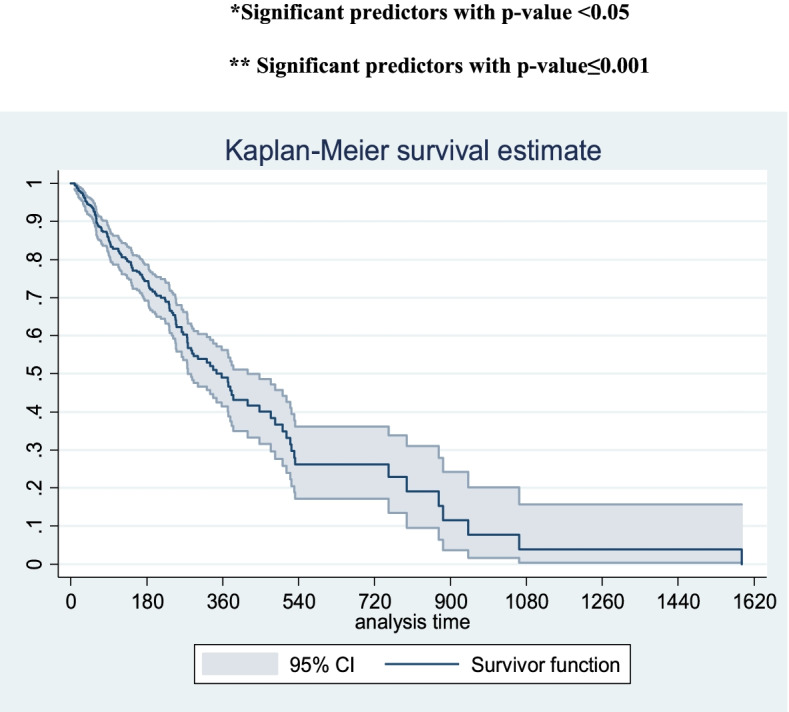
Overall Kaplan-Meier survival curve with 95% confidence interval showing the survival time of chronic kidney disease patients on hemodialysis in Amhara region, Ethiopia, 2021

The overall Kaplan-Meier survival curve also shows a steep decrease in the survival rate of CKD patients on hemodialysis in the first 540 days (where the highest number of deaths occurs), then the curve becomes steady until 720 days and it steadily declines afterward as shown in Fig. 1.

### Predictors of mortality among end-stage renal disease patients on hemodialysis

Variables that had a *P*-value < 0.25 in the bivariable cox regression were residence, CKD secondary to hypertension, CKD secondary to glomerulonephritis, CKD secondary to HIV, vascular access, frequency of dialysis per week, duration of dialysis per session, adequacy of dialysis, and age of the patients.

The proportional hazard assumption was checked statistically using the global goodness-of-fit test using Schoenfeld residuals. The proportional hazard assumption was fulfilled with a global test *P*-value of 0.5661. The assumption was also checked for each predictor with minimum and maximum *p* values of 0.0825 and 0.8873 respectively (Table 6).

**Table 6:** Proportional hazard assumption output table of the variables included in the multivariable analysis among ESRD patients on hemodialysis in Amhara regional state, Ethiopia, 2016-21

Variable categories		Rho	chi2	Df	Prob > chi2
Residence	Urban			1	0.8780
	Rural	0.01153	0.02	1	
Hypertension	No			1	
	Yes	-0.11380	3.02	1	0.0825
Glomerulonephritis	No			1	
	Yes	0.01305	0.02	1	0.8873
HIV	No			1	
	Yes	-0.07261	0.83	1	0.3457
Vascular access	Fistula			1	
	Catheter	-0.06690	0.73	1	0.3932
	Graft	0.05156	0.48	1	0.4895
Frequency per week	Once			1	
	Twice	0.01067	0.02	1	0.8889
	three times	0.05077	0.43	1	0.5125
Duration per session	< 3 h.			1	
	3 and 1/2 Hrs.	0.02628	0.11	1	0.7349
	4 h	0.01825	0.05	1	0.8237
Adequacy	< 1.2 kt/v			1	
	> 1.2 kt/v	0.08118	1.52	1	0.2170
Age		0.12260	2.53	1	0.1115
global test			10.57	12	0.5661

Among the variables which were included in the multivariable Cox proportional hazard model five variables, residence, hypertension as a cause of CKD, HIV as a cause of CKD, central venous catheter, and 4 h of duration of dialysis per session were significantly (*P*-value < 0.05) associated with mortality among ESRD patients on hemodialysis after adjusting for the other variables.

The risk of mortality among patients who live in rural residences was 1.48 times (95%CI (1.04, 2.12)) higher than patients who live in urban residences. The risk of death was 1.49 times (95%CI (1.01, 2.23)) higher among patients with ESRD secondary to hypertension than patients with other causes. Those patients with ESRD secondary to HIV had 2.22 times (95%CI (1.41, 3.51)) higher risk of mortality than patients with other causes of ESRD. Patients who used central venous catheters had 3.15 times (95%CI (2.08, 4.77)) higher risk of death than those patients who use arteriovenous fistula as their vascular access.

The risk of mortality among patients who stayed 4 h on hemodialysis decreased by 57% (95%CI (0.23, 0.80)) than those patients who stay 3 h on hemodialysis per session (Table 7).

**Table 7:** Results from bivariable and multivariable Cox proportional hazard model analysis of ESRD patients on hemodialysis in Amhara regional state, Ethiopia, 2016-21 (*n* = 436)

Variable categories		Status		CHR (95% CI)	AHR (95% CI)
		Death	Censored		
Residence	Urban	90	192	1	1
	Rural	63	91	1.67 (1.20, 2.31)	1.48 (1.04, 2.12) *
Hypertension	No	81	130	1	1
	Yes	72	153	0.76 (0.55, 1.05)	1.49 (1.01, 2.23) *
Glomerulonephritis	No	102	197	1	**1**
	Yes	51	86	1.39 (0.99, 1.95)	1.09 (0.72, 1.65)
HIV	Yes	123	266	1	1
	No	30	17	2.12 (1.41, 3.15)	2.22 (1.41, 3.51) **
Vascular access	Fistula	61	160	1	1
	Catheter	76	95	3.16 (2.21, 4.51)	3.15 (2.08, 4.77) **
	Graft	16	28	2.02 (1.15, 3.56)	1.66 (0.88, 3.13)
Frequency per week	Once	14	18	1	1
	Twice	105	226	0.56 (0.32, 0.97)	0.68 (0.37, 1.26)
	three times	34	39	1.15 (0.61, 2.15)	1.11 (0.57, 2.14)
Duration per session	< 3 h.	14	9	1	1
	3&1/2 Hrs.	13	22	0.57 (0.26, 1.20)	0.57(0.26, 1.26)
	4 h	126	252	**0.35 (0.20, 0.62)**	0.43 (0.23, 0.80) *
Adequacy	< 1.2 kt/v	33	41	1	1
	> 1.2 kt/v	120	242	0.58 (0.39, 0.86)	1.37 (0.83, 2.27)
Age				1.01 (1.00, 1.02)	1.01 (0.99, 1.02)

*Significant predictors with *p*-value < 0.05

** Significant predictors with *p*-value ≤ 0.001

## Discussion

Overall, this study shows that the overall survival proportion of ESRD patients on hemodialysis was 64.9% which was lower than that of the studies done in Ethiopia Adama, Saint Gabrial, and Ayider [15–17]. This result might be due to the inclusion of ESRD patients who were on hemodialysis in the first three months of hemodialysis in the current study [19].

The result was also lower than studies done in England, Scotland, the United Kingdom, India, and South Africa [20–23]. The low survival rate of the study is likely attributed to late presentation, frequent dialysis discontinuation, suboptimum dialysis quality, poor quality of care before referral, financial constraints, and the lack of national screening and management programs for ESRD in most of sub-Saharan Africa including Ethiopia [24, 25].

Even though, the median survival time of the study was 345 days which was consistent with the study conducted in Ayider, the incidence of death in the current study was 1.89 per 1000 person-days which was higher than studies conducted in Ethiopia, Ayider comprehensive specialized hospital and Ghana [7, 15]. This result might be explained by the relatively longer follow-up time of the current study [19, 21].

On the other hand, the median survival time was higher than in a study conducted at Black lion hospital [14]. This result might be due to the time difference between the studies and improved hemodialysis service quality and increased access to hemodialysis in Ethiopia throughout the years [18].

The risk of mortality among patients who live in rural residences was 1.48 times higher than their counterparts which were in agreement with a study conducted in the United States [26]. The high mortality among rural residents might be attributed to distance from the dialysis centers, less affordability, low accessibility, low knowledge about ESRD management due to decreased access to health education, and high susceptibility to infections from their day to day activities among patients from rural residents when compared with patients who live in urban residents [27].

The risk of death was 1.49 times higher among patients with ESRD secondary to hypertension than patients with no hypertension. The result was in line with studies conducted in China, Korea, Cameron, and the United States [28–31]. Reduced compliance, side effects, and financial costs among hypertensive patients might impact hemodialysis effectiveness, systolic blood pressure, and diastolic blood pressure along with traditional risk factors for cardiovascular disease, which are associated with end-organ damage, including vascular stiffness and mortality of patients on dialysis [32].

Patients whose primary cause of nephropathy was HIV had 2.22 times increased risk of mortality than those patients with a primary cause other than HIV. This result was consistent with a study conducted in Ethiopia, Ayider hospital [15]. This similarity might be attributed to the compromised immune status of HIV-positive patients and the use of vascular access different from AVF, since they are easily susceptible to vascular site infection, bacteremia, and opportunistic infections [33, 34].

Patients who used central venous catheters had 3.15 times more risk of death than those patients who use arteriovenous fistula as their vascular access. The result was supported by studies done in Tehran, Japan, Madrid, and Birmingham [35–38]. This result might implicate that patients with vascular access to central venous catheters were commonly prone to prolonged hospitalizations, and complications due to serious invasive infections [39].

This study shows that, compared with patients who stay 3 h on hemodialysis per session, staying 4 h on hemodialysis decreases the risk of mortality by 57%, which is consistent with studies conducted in Washington, Australia, New Zealand, and Saint Gebrial [17, 40–42]. This might be due to a shorter duration of hemodialysis being more likely to result in complications, increased hospitalizations, and increased mortality [43].

The main limitation of the study was that it does not address socioeconomic factors (such as the income of the patients) that might affect the survival of the patients and that might also be a possible confounder. Since the factors that affect the mortality of patients who were on hemodialysis were not fully included because of the incompleteness of the records, the associations observed in the study might be potentially confounded by underlying factors.

## Conclusions

There was a significantly high mortality rate and relatively lower median survival time of chronic kidney disease patients on hemodialysis when compared to middle- and high-income countries. The highest mortality occurred during the first three months after the initiation of hemodialysis. Hence Ethiopian ministry of health shall work towards addressing hemodialysis services for patients who live in rural residences and has low socioeconomic status.

Researchers should consider conducting a prospective follow-up study triangulated with qualitative data to address the behavioral characteristics, socioeconomic status, and body mass index of the patients.

## Data Availability

The datasets used and/or analyzed during the current study are available from the corresponding author on reasonable request. The datasets generated during and analyzed during the current study are not publicly available because the hospitals do not permit the researchers to share the datasets publicly.

## Abbreviations

AHR: Adjusted Hazard Ratio
AVF: Arteriovenous Fistula
BDU: Bahir Dar University
CKD: Chronic Kidney Disease
CVC: Central Venous Catheter
CVD: Cardio Vascular Disease
ESRD: End-Stage Renal Disease
FHCSH: Felege Hiwot Comprehensive Specialized Hospital
GFR: Glomerular Filtration Rate
HIVAN: Human Immune Virus Associated Nephropathy
RRT: Renal Replacement Therapy

## Declarations

Since only secondary data were utilized for the study, there was no direct contact with the patients, and a phone call to confirm the patient’s outcome was made to their close families. Bahir Dar University College of medicine and health science Institutional Review Board (IRB) approved the use of patient medical records and confirmed the outcome of the patients using a phone call with the permission of the hospitals with a letter written on February 26, 2021, with a protocol number of 082/2021. Since most of the patients could not be accessed (lost to follow-up or dead) to take informed consent from the patients, the consent was taken from their family members, and for those patients who were on follow-up during the study, informed consent was obtained.

Confidentiality of the information was secured throughout the study by excluding names and patient record numbers as identification in the data extraction form and the data were used only for the proposed study.

The data for this retrospective follow-up were collected following the Declaration of Helsinki. I confirm that all methods were performed per the relevant guidelines and regulations by including a statement in the “ethics approval and consent to participate” section under ‘Declarations’ to this effect.

## References

[1] Anand S, Bitton A, Gaziano T (2013). The gap between estimated incidence of end-stage renal disease and use of therapy. PLoS One.

[2] Dr. A. What are Survival Rates for Patients on Dialysis? Renal Fellow Network. September 19, 2018.

[3] Liyanage T, Ninomiya T, Jha V, Neal B, Patrice HM, Okpechi I (2015). Worldwide access to treatment for end-stage kidney disease: a systematic review. Lancet.

[4] Xie Y, Bowe B, Mokdad AH, Xian H, Yan Y, Li T (2018). Analysis of the Global Burden of Disease study highlights the global, regional, and national trends of chronic kidney disease epidemiology from 1990 to 2016. Kidney Int.

[5] Davids MR, Eastwood JB, Selwood NH, Arogundade FA, Ashuntantang G, Benghanem Gharbi M (2016). A renal registry for Africa: first steps. Clin Kidney J.

[6] McQuillan R, Trpeski L, Fenton S, Lok CE. Modifiable Risk Factors for Early Mortality on Hemodialysis. Int J Nephrol. 2012;2012:435736. 10.1155/2012/435736 PMC3409533 22888426

[7] Eghan BA, AMOAKO-ATTA K, Kankam CA, NSIAH‐ASARE A (2009). Survival pattern of hemodialysis patients in Kumasi, Ghana: a summary of forty patients initiated on hemodialysis at a new hemodialysis unit. Hemodialysis Int.

[8] Moeller S, Gioberge S, Brown G (2002). ESRD patients in 2001: global overview of patients, treatment modalities and development trends. Nephrol Dialysis Transplant.

[9] Girum T, Mesfin D, Bedewi J, Shewangizaw M. The burden of noncommunicable diseases in Ethiopia, 2000–2016: analysis of evidence from global burden of disease study 2016 and global health estimates 2016. Int J Chronic Dis. 2020;2020. 10.1155/2020/3679528 PMC7053448 32149073

[10] Paltiel O, Berhe E, Aberha AH, Tequare MH, Balabanova D (2020). A public–private partnership for dialysis provision in Ethiopia: a model for high-cost care in low-resource settings. Health Policy Plan.

[11] Shimels T, Bilal AI (2019). Hemodialysis or transplantation for Ethiopia: a cost utility analysis. AABSc.

[12] Tadesse H, Gutema H, Wasihun Y, Dagne S, Menber Y, Petrucka P, et al. Lived Experiences of Patients with Chronic Kidney Disease Receiving Hemodialysis in Felege Hiwot Comprehensive Specialized Hospital, Northwest Ethiopia. Int J Nephrol. 2021;2021. 10.1155/2021/6637272 PMC8410445 34484835

[13] Kassa DA, Mekonnen S, Kebede A, Haile TG. Cost of Hemodialysis Treatment and Associated Factors Among End-Stage Renal Disease Patients at the Tertiary Hospitals of Addis Ababa City and Amhara Region, Ethiopia. Clinicoeconomics Outcomes Res. CEOR. 2020;12:399. 10.2147/CEOR.S256947 PMC7419632 32821136

[14] Shibiru T, Gudina EK, Habte B, Deribew A, Agonafer T (2013). Survival patterns of patients on maintenance hemodialysis for end stage renal disease in Ethiopia: summary of 91 cases. BMC Nephrol.

[15] Belachew AB, Mohammed AN, Kahsay AB. Determinants of Survival among Hemodialysis Patients at Ayder Comprehensive Specialized Hospital, Tigray, North Ethiopia: A Retrospective Cohort Study. Research & Reviews. J Oncol Hematol. 2019;7(3):22 – 9.

[16] Hussein M, Muleta G, Seyoum D, Kifle D, Bedada D (2017). Survival Analysis of Patients with End Stage Renal Disease the Case of Adama Hospital, Ethiopia. Clin Med Res.

[17] Mequanent Wale M, Birahan KA, Chekole DM, Derso EA (2020). Determinants of Overall Survival of Kidney Failure for Patients receiving Dialysis in Saint Geberial General Hospital, Addis Ababa, Ethiopia. J Kidney.

[18] Bekele M. Historical Milestones of Renal Replacement Therapy in Ethiopia. Ethiopian Med J. 2020.

[19] Jardine T, Wong E, Steenkamp R, Caskey FJ, Davids MR (2020). Survival of South African patients on renal replacement therapy. Clin Kidney J.

[20] UK Renal Registry 21st Annual Report – Data to 31/12/2017. Bristol UU, 2019.

[21] Davids MR, Jardine T, Marais N, Jacobs JC, Sebastian S. south African renal registry Annual report 2018. Afr J Nephrol. 2020;23(1):185–96.

[22] Halle MP, Takongue C, Kengne AP, Kaze FF, Ngu KB (2015). Epidemiological profile of patients with end stage renal disease in a referral hospital in Cameroon. BMC Nephrol.

[23] Chandrashekar A, Ramakrishnan S, Rangarajan D (2014). Survival analysis of patients on maintenance hemodialysis. Indian J Nephrol.

[24] Kaze FF, Ashuntantang G, Kengne AP, Hassan A, Halle MP, Muna W (2012). Acute hemodialysis complications in end-stage renal disease patients: The burden and implications for the under‐resourced S ub‐S aharan A frican health systems. Hemodialysis Int.

[25] Ashuntantang G, Osafo C, Olowu WA, Arogundade F, Niang A, Porter J (2017). Outcomes in adults and children with end-stage kidney disease requiring dialysis in sub-Saharan Africa: a systematic review. Lancet Global Health.

[26] Maripuri S, Arbogast P, Ikizler TA, Cavanaugh KL (2012). Rural and micropolitan residence and mortality in patients on dialysis. Clin J Am Soc Nephrol.

[27] Muiru AN, Charlebois ED, Balzer LB, Kwarisiima D, Elly A, Black D (2020). The epidemiology of chronic kidney disease (CKD) in rural East Africa: A population-based study. PLOS ONE.

[28] Yao X, Lei W, Shi N, Lin W, Du X, Zhang P (2020). Impact of initial dialysis modality on the survival of patients with ESRD in eastern China: a propensity-matched study. BMC Nephrol.

[29] Halle MP, Ashuntantang G, Kaze FF, Takongue C, Kengne A-P (2016). Fatal outcomes among patients on maintenance haemodialysis in sub-Saharan Africa: a 10-year audit from the Douala General Hospital in Cameroon. BMC Nephrol.

[30] Mathew A, Obi Y, Rhee CM, Chen JL, Shah G, Lau W-L (2016). Treatment frequency and mortality among incident hemodialysis patients in the United States comparing incremental with standard and more frequent dialysis. Kidney Int.

[31] van Manen JG, van Dijk PC, Stel VS, Dekker FW, Clèries M, Conte F (2007). Confounding effect of comorbidity in survival studies in patients on renal replacement therapy. Nephrol Dialysis Transplant.

[32] Li Z, Lacson E, Lowrie EG, Ofsthun NJ, Kuhlmann MK, Lazarus JM (2006). The epidemiology of systolic blood pressure and death risk in hemodialysis patients. Am J Kidney Dis.

[33] Kim S, Jeong JC, Ahn SY, Doh K, Jin D-C, Na KY (2019). Time-varying effects of body mass index on mortality among hemodialysis patients: Results from a nationwide Korean registry. Kidney Res Clin Pract.

[34] Halle MP, Edjomo AM, Fouda H, Djantio H, Essomba N, Ashuntantang GE (2018). Survival of HIV infected patients on maintenance hemodialysis in Cameroon: a comparative study. BMC Nephrol.

[35] Soleymanian T, Sheikh V, Tareh F, Argani H, Ossareh S (2017). Hemodialysis vascular access and clinical outcomes: an observational multicenter study. J Vascular Access.

[36] Ong S, Barker-Finkel J, Allon M (2013). Long-term outcomes of arteriovenous thigh grafts in hemodialysis patients: a comparison with tunneled dialysis catheters. Clin J Am Soc Nephrol.

[37] Gil Giraldo Y, Muñoz Ramos P, Ruano P, Quiroga B. Vascular access-related mortality in hemodialysis patients during and after hospitalization. Therapeutic Apheresis Dialysis. 2020;24(6):688–94. PubMed PMID: 31989776. Epub 2020/01/29. eng. 10.1111/1744-9987.13479 31989776

[38] Yu Y, Xiong Y, Zhang C, Fu M, Li Y, Fu P (2020). Vascular Access Type Was Not Associated with Mortality and the Predictors for Cardiovascular Death in Elderly Chinese Patients on Hemodialysis. Blood Purification.

[39] Zhang HH, Cortés-Penfield NW, Mandayam S, Niu J, Atmar RL, Wu E (2019). Dialysis Catheter–related bloodstream infections in patients receiving hemodialysis on an emergency-only basis: a retrospective cohort analysis. Clin Infect Dis.

[40] Swaminathan S, Mor V, Mehrotra R, Trivedi AN (2017). Initial session duration and mortality among incident hemodialysis patients. Am J Kidney Dis.

[41] Marshall M, Byrne B, Kerr P, McDonald SP (2006). Associations of hemodialysis dose and session length with mortality risk in Australian and New Zealand patients. Kidney Int.

[42] Lok CE, Foley R (2013). Vascular access morbidity and mortality: trends of the last decade. Clin J Am Soc Nephrol.

[43] Rivara MB, Adams SV, Kuttykrishnan S, Kalantar-Zadeh K, Arah OA, Cheung AK (2016). Extended-hours hemodialysis is associated with lower mortality risk in patients with end-stage renal disease. Kidney Int..

